# Legal and ethical aspects of organ donation and transplantation

**DOI:** 10.4103/0970-1591.56203

**Published:** 2009

**Authors:** Sunil Shroff

**Affiliations:** Department of Urology and Renal Transplantation, Sri Ramachandra Medical College & Research Institute, Porur, Chennai 600 116, India

**Keywords:** Cadaver transplantation, ethics in transplantation, related donor

## Abstract

The legislation called the Transplantation of Human Organ Act (THO) was passed in India in 1994 to streamline organ donation and transplantation activities. Broadly, the act accepted brain death as a form of death and made the sale of organs a punishable offence. With the acceptance of brain death, it became possible to not only undertake kidney transplantations but also start other solid organ transplants like liver, heart, lungs, and pancreas. Despite the THO legislation, organ commerce and kidney scandals are regularly reported in the Indian media. In most instances, the implementation of the law has been flawed and more often than once its provisions have been abused. Parallel to the living related and unrelated donation program, the deceased donation program has slowly evolved in a few states. In approximately one-third of all liver transplants, the organs have come from the deceased donor program as have all the hearts and pancreas transplants. In these states, a few hospitals along with committed NGOs have kept the momentum of the deceased donor program. The MOHAN Foundation (NGO based in Tamil Nadu and Andhra Pradesh) has facilitated 400 of the 1,300 deceased organ transplants performed in the country over the last 14 years. To overcome organ shortage, developed countries are re-looking at the ethics of unrelated programs and there seems to be a move towards making this an acceptable legal alternative. The supply of deceased donors in these countries has peaked and there has been no further increase over the last few years. India is currently having a deceased donation rate of 0.05 to 0.08 per million population. We need to find a solution on how we can utilize the potentially large pool of trauma-related brain deaths for organ donation. This year in the state of Tamil Nadu, the Government has passed seven special orders. These orders are expected to streamline the activity of deceased donors and help increase their numbers. Recently, on July 30, 2008, the Government brought in a few new amendments as a Gazette with the purpose of putting a stop to organ commerce. The ethics of commerce in organ donation and transplant tourism has been widely criticized by international bodies. The legal and ethical principles that we follow universally with organ donation and transplantation are also important for the future as these may be used to resolve our conflicts related to emerging sciences such as cloning, tissue engineering, and stem cells.

## INTRODUCTION

Kidney transplants in India first started in the 1970s and since that time, India has been a leading country in this field on the Asian sub-continent. The evolutionary history of transplants in the last four decades has witnessed a different facet of transplant emerging in each decade. The first 10 years were spent mastering the surgical techniques and immune-suppression. Its success resulted in a phenomenal rise in the numbers of transplants in the next 10 years and unrelated kidney donation from economically weaker sections started taking place with commerce in organ donation becoming an acceptable integral part of the program. After this was accepted, the ethics of transplants in India has always been on a slippery slope and all kinds of nefarious activities were accepted as normal practice. The general dictum was “when you can buy one why donate?” The next 10 years saw an outcry from the physicians of the western world at the growing numbers of these exploitative transplants being done in India. There were also protests from many sections in India. The pressure on the Government saw the passing of the Transplantation of Human Organ Act (THO) legislation that made unrelated transplants illegal and deceased donation a legal option with the acceptance of brain death.[1] Overcoming organ shortage by tapping into the pool of brain-dead patients was expected to curb the unrelated transplant activity. The last decade has seen the struggle of the deceased donation program evolve in India. Simultaneously, it has witnessed the living donation program being marred with constant kidney scandals. In most instances, the donor accused the recipient or the middle man of having not compensated them with the promised sum. It also saw liver, heart, and pancreas transplants from deceased donors. Although the history of cadaver transplants in India is recent, the first attempts to use a cadaver donor's kidney were undertaken in 1965 in Mumbai. The author describes the medical and social problems they faced. The medical problems included technical difficulties in engrafting, immunological problems, and infection. However, it was the hostile reaction from some members of the medical profession and the general public that was a more daunting task to tackle. The whole process was described by some as neo-cannibalism. This was a setback for the cadaver program for not only Mumbai but also rest of the country.[2]

In India, despite the THO act, neither has the commerce stopped nor have the number of deceased donors increased to take care of organ shortage.[3] The concept of brain death has never been promoted or widely publicized. Most unrelated transplants currently are being done under the cloak of legal authority from an authorization committee. The few deceased donations that are taking place are due to the efforts of a few Non Government Organizations (NGO) or hospitals that are highly committed to the cause. Recently, the government has come under much criticism by the public and media and has added a few legislations in the form of a Gazette to curb the illegal unrelated donation activities and has tried to plug the loopholes in the THO act.[4] To a large extent, the failure of the THO act has been because of the way it has been interpreted and implemented by authorities and hospitals.

## THE LAW AND RULES GOVERNING ORGAN DONATION AND TRANSPLANTATION IN INDIA

The main provisions of the THO act and the newly passed Gazette by the Government of India include the following:

1. For living donation - it defines who can donate without any legal formalities. The relatives who are allowed to donate include mother, father, brothers, sisters, son, daughter, and spouse. Recently, in the new Gazette grandparents have been included in the list of first relatives. The first relatives are required to provide proof of their relationship by genetic testing and/or by legal documents. In the event of there being no first relatives, the recipient and donor are required to seek special permission from the government appointed authorization committee and appear for an interview in front of the committee to prove that the motive of donation is purely out of altruism or affection for the recipient.
2. Brain-death and its declaration - brain death is defined by the following criteria: two certifications are required 6 hours apart from doctors and two of these have to be doctors nominated by the appropriate authority of the government with one of the two being an expert in the field of neurology.
3. Regulation of transplant activities by forming an Authorization Committee (AC) and Appropriate Authority (AA.) in each State or Union Territory. Each has a defined role as follows:
  - Role of Authorization Committee (AC) - The purpose of this body is to regulate the process of authorization to approve or reject transplants between the recipient and donors other than a first relative. The primary duty of the committee is to ensure that the donor is not being exploited for monetary consideration to donate their organ. The joint application made by the recipient and donor is scrutinized and a personal interview is essential to satisfy to the AC the genuine motive of donation and to ensure that the donor understands the potential risks of the surgery. Information about approval or rejection is sent by mail to the concerned hospitals. The decision to accept or reject a donor is governed by Sub Clause (3), Clause 9 of Chapter II of the THO act.
  - Role of Appropriate Authority (AA): The purpose of this body is to regulate the removal, storage, and transplantation of human organs. A hospital is permitted to perform such activities only after being licensed by the authority. The removal of eyes from a dead body of a donor is not governed by such an authority and can be done at other premises and does not require any licensing procedure. The powers of the AA include inspecting and granting registration to the hospitals for transplant surgery, enforcing the required standards for hospitals, conducting regular inspections of the hospitals to examine the quality of transplantation and follow-up medical care of donors and recipients, suspending or canceling the registrations or erring hospitals, and conducting investigations into complaints for breach of any provisions of the Act. The AA issues a license to a hospital for a period of 5 years at a time and can renew the license after that period. Each organ requires a separate license.

### Application Forms

The Transplantation of Human Organ Act clearly lays out various procedures; for this purpose, it has thirteen different forms [Table 1]. The Central Government has amended the Transplantation of Human Organs Act, 1994 (42 of 1994) to include certain changes called the Transplantation of Human Organs Rules, 1995 (GSR NO. 51(E), dr. 4-2-1995) [As amended vide GSR 571(E), dt.31-7-2008]. Given below are important excerpts from the rules.

### Authority for removal of human organ

Any donor may authorize the removal, before his death, of any human organ of his body for therapeutic purposes as specified in Forms 1(A), 1(B), and 1(C). The new forms have been made more comprehensive and are to be submitted with proof of identity and address, marriage registration certificate, family photographs, etc. with attestation by a Notary Public.

The gazette states that before removing a human organ from the body of a donor before his death, a medical practitioner should satisfy himself that the donor has given authorization in Form 1(A) if the relative is a close relative i.e., a mother, father, brother, sister, son, or daughter. Form 1(B) is used for a spouse and Form 1(C) is used for other relatives. He should also confirm the following:

- The donor is in a proper state of health and is fit to donate the organ. The registered medical practitioner should then sign a certificate as specified in Form 2.
- The donor is a close relative of the recipient as certified in Form 3 and has signed Form 1(A).
- The donor has submitted an application in Form 10 jointly with the recipient and the proposed donation has been approved by the concerned competent authority. The relationship between the donor and recipient also needs to be examined to the satisfaction of the Registered Medical Practitioner in charge of the transplant center.
  - In the case of the recipient being a spouse of the donor, the donor has given a statement to the effect that they are so related by signing a certificate in Form 1(B) and has submitted an application in Form 10 jointly with the recipient and the proposed donation has been approved by the concerned competent authority.
  - In the case of a donor who is other than a close relative, the donor has signed Form 1(C), submitted an application in Form 10 jointly with the recipient, and permission from the Authorization Committee for the donation has been obtained.

A registered medical practitioner shall, before removing a human organ from the body of a person after his death, confirm the following:

- The donor had, in the presence of two or more witnesses (at least one of whom is a close relative of the recipient), unequivocally authorized as specified in Form 5 before his death, the removal of the human organ of his body after his death for therapeutic purposes and there is no reason to believe that the donor had subsequently revoked the authority.
- The person lawfully in possession of the dead body has signed a certificate as specified in Form 6.

A registered medical practitioner shall, before removing a human organ from the body of a person in the event of brain-stem death, confirm the following:

- A certificate as specified in Form 8 has been signed by all the members of the Board of Medical Experts.
- In the case of brain-stem death of a person of less than 18 years of age, a certificate specified in Form 8 has been signed by all the members of the Board of Medical Experts and an authority as specified in Form 9 has been signed by either of the parents the person.

### Working Guidelines for the Authorization Committee

The new gazette clearly lays down the following guidelines:

1. Where the proposed transplant is between persons related genetically (close relative, i.e., mother, father, brother, sister, son, or daughter above the age of 18 years old), the following shall be evaluated:

- Results of tissue typing and other basic tests
- Documentary evidence of relationship e.g., relevant birth certificates and marriage certificate
- Documentary evidence of identity and residence of the proposed donor e.g., Ration Card or Voters Identity Card, Passport, Driving License, PAN Card, or Bank Account and family photograph depicting the proposed donor and the proposed recipient along with another near relative
- If the relationship is not conclusively established after evaluating the above evidence, direct further medical tests may be given as described follows.
  – Test for Human Leukocyte Antigen (HLA), human leukocyte antigen-B alleles to be performed by the serological and /or polymerase chain reaction (PCR) based Deoxyribonucleic Acid (DNA) methods
  – Test for human leukocyte antigen-Dr beta genes to be performed using PCR-based DNA methods.

Tests shall be done from a laboratory accredited with National Accreditation Board for Laboratories (NABL). When the tests referred to above do not establish a genetic relationship between the donor and the recipient, the same tests should be performed on both or at least one parent, preferably both parents. If parents are not available, the same tests should be performed on relatives of donor and recipient that are available and are willing to be tested failing which, the genetic relationship between the donor and the recipient will be deemed to have not been established.

When the proposed transplantation is between a married couple, the Registered Medical Practitioner i.e., the person in charge of the transplant center must evaluate the fact and duration of marriage (marriage certificate, marriage and family photographs, birth certificate of children containing particulars of parents). When the proposed donor or recipient or both are not Indian Nationals/citizens whether close relatives or otherwise, the AC shall consider all such requests. A senior Embassy official of the country of origin has to certify the relationship between the donor and the recipient. When the proposed donor and the recipient are not close relatives, the Authorization Committee shall evaluate that there is no commercial transaction between the recipient and the donor and the following shall specifically be assessed:

- An explanation of the link between them and the circumstances that led to the offer being made
- Reasons why the donor wishes to donate
- Documentary evidence of the link, e.g., proof that they have lived together
- Old photographs showing the donor and recipient together
- There is no middleman or tout involved
- The financial status of the donor and the recipient is probed by asking them to give appropriate evidence of their vocation and income for the previous three financial years. Any gross disparity between the status of the two must be evaluated with the objective of preventing commercial dealing.
- The donor is not a drug addict or known person with criminal record
- The next of kin of the proposed unrelated donor is interviewed regarding awareness about his or her intention to donate an organ, the authenticity of the link between the donor and the recipient and the reasons for donation.

The AC should state in writing its reason for rejecting or approving the application of the proposed donor and all approvals should be subject to the following conditions:

- The approved proposed donor would be subjected to all medical tests as required at relevant stages to determine his biological capacity and compatibility to donate the organ in question.
- Psychiatrist's clearance in such cases is deemed mandatory to certify the donor's mental condition, awareness, absence of any overt or latent psychiatric disease, and ability to give free consent.
- All prescribed forms have been completed by all relevant persons involved in the process of the transplantation.
- All interviews should be video recorded.

The AC is required to take a final decision within 24 hours of the meeting for grant of permission or rejection for transplant. Every authorized transplantation center must have its own website. The decision of the AC should be displayed on the notice board of the hospital immediately and on the website of the hospital or institution within 24 hours of making the decision.

### Guidelines for composition of the AC

There shall be one State Level AC. It will provide approval or a no objection certificate to the donor and recipient to establish legal and residential status in a particular state. Additional ACs may be set up at various levels as per the requirements as follows:

- No member from the transplant team of the institution should be a member of the respective AC.
- The AC should be hospital-based in metros and big cities if the number of transplants exceeds 25 in a year at the respective transplantation centers. In small towns, there shall be state or district level committees if transplants are less than 25 in a year in the respective districts.

### Composition of a hospital-based AC

- Medical Director or Medical Superintendent of the Hospital
- Two senior medical practitioners from the same hospital who are not part of the transplant team
- Two members of high integrity, social standing, and credibility
- Secretary (Health) or nominee and Director Health Services or nominee

### Composition of State or District Level ACs

- Medical Practitioner officiating as Chief Medical Officer or any other equivalent post in a main/major government hospital of the district
- Two senior medical practitioners who are residing in the concerned district and who are not part of any transplant team
- Two senior citizens of high reputation and integrity residing in the same district
- Secretary (Health) or nominee and Director of Health Services or nominee

## INTERPRETATION OF LAW

To a large extent, the interpretation of the THO act by the AC and the registered medical practitioners has been flawed. To a large extent this has been addressed in the current Gazette. However, this Gazette has to be passed by the state governments before it becomes compulsory for the hospitals to follow the ruling.

The provisions available in Sub Clause (3), Clause 9 of Chapter II of the THO act states “If any donor authorizes the removal of any of his human organs before his death under sub-section (1) of Section 3 for transplantation into the body of such recipient, not being a near relative as is specified by the donor, by reason of affection or attachment towards the recipient or for any other special reasons, such human organ shall not be removed and transplanted without the prior approval of the Authorization Committee” has been misused or misinterpreted by one and all over the years, since the act was passed.

### The Transplant Clinicians Interpretation

The clinicians wonder if the law itself provides a clause to help people whose own family members refuse to donate or those who do not have a fit or matching donor, why should they refuse any arrangement between the donor and recipient? To them the plight of the recipient overrules all objections. They also argue that it is difficult for them to understand the so-called true affection. They feel the onus of responsibility to find true affection and relationships rests under the purview of the government AC.

### Misuse by the paid donor

A patient whose kidney has failed uses this clause to find an instant affection in a stranger who is willing to donate his/her organ for money but they deny any such information to the AC. Later, the same donor makes a claim to the police or the media that they were duped into the donation process and not paid the promised sum for the organ. The affection in these instances, which they expressed for the recipient in front of the AC has no meaning or relevance. The police having no knowledge that the act of donation for money is illegal instantly pulls up the middleman or doctor or the hospital. Occasionally, when there is a media expose, the authorization committee in a knee-jerk reaction, tightens its regulations and stops clearing even genuine cases. For the past 3 years, the AC in Tamil Nadu videotaped all the interviews so that these videos can be used as an evidence later if necessary.

### The authorization committee's interpretation

When presented provisions of the law, the AC concludes that if the recipient and donor pledge affection in front of them they should not object unless there is a complaint or some gross oversight. They also believe that since the doctor himself has sent such a case to the committee, they need verify such claims. The majority of applications to the AC are usually accepted. Most unrelated donations occur when the donor expresses their true affection for the recipient in front of the AC. Between 1995 and 2002, there were about 5,000 cases interviewed by the AC in Tamil Nadu with a rejection rate of less than 5%. In another memo issued by the Department of Health of Tamil Nadu, it indicated that during January 2000 to May 2002 they had approved 1,559 unrelated transplants out of the 1,868 applications received. The scenario in other states in the country where transplants are done is similar to Tamil Nadu.

As per the law, any person who is aggrieved with the order of the AC is allowed to make an appeal within 30 days of the issue of the order to the State government. In, B.L. Nagaraj and others *vs*. Kantha and others, the prospective recipient filed a writ petition before the High Court of Karnataka against the order of the AC that rejected the application for organ donation by the sister-in-law of the recipient on the grounds that close relatives were not considered donors. The High Court while allowing the writ petition held:

“There is no provision in the Act which prohibits the person who is not a 'near relative' by definition, from donating his kidney merely because the 'near relative' has not been considered as donors by the family for kidney transplantation. The Committee has misdirected itself in this regard while refusing permission to the petitioners.”

“The Committee would ascertain from the second petitioner whether she would be donating the kidney out of ‘affection and attachment’. The donors relationship with the recipient, period of acquaintance and the degree of association, reciprocity of feelings, gratitude and other human bonds are perhaps some of the factors which would sustain ‘affection and attachment’ between two individuals. The committee has to ensure that the human organ does not become an article of commerce. The main thrust of the act is against commercial dealings in human organs.”

The problem has been on how to use Sub-Clause (3), Clause 9 of Chapter II of the THO act and how to protect the exploitative element in the word affection. In 1997, Dr. M.K. Mani, a leading Nephrologist in Chennai, summarized the above very well when he wrote: “The stalwarts of the unrelated live donor program continue to do as many transplants as they did before the Legislative Assembly of Tamil Nadu adopted the Act. What is more, they do them with the seal of approval from the Authorization Committee and are therefore a very satisfied lot. The law, which was meant to prohibit commercial dealings in human organs, now provides protection for those very commercial dealings.” Dr. Mani's article is titled ‘The Law is an Ass’.[5]

After a major kidney racket in Tamil Nadu, the Department of Health issued a notification in form of a ‘Government Order’ trying to absolve all responsibilities to prove relationship or any possibility of commerce with AC. It categorically stated that the responsibility to prove such a relationship was solely on the doctors of the hospital who signed the document to request for an interview. However, this was against what the THO act itself states and the role it defines for authorization committee. When the legal standing of the order was questioned, the order was withdrawn. The new Gazette now requires videotaping of the whole proceedings of the interview. In addition, it also gives guidelines to the AC and clearly states that there should be no tout or middleman with the donor having to provide an explanation of why he wishes to donate with documentary proof of having lived together (old Photographs) and information about his vocation with financial statements from the previous 3 years. Taking away the ambiguity of the term affection and giving it the seriousness it deserves may go some way in preventing the sale of kidneys.

## ETHICS OF ORGAN SALE

The presence of a growing middle class, the lack of a national health insurance scheme, the growing disparity between the rich and poor, and to some extent the presence of technology in the country makes the process of commodification of organs a simple, quick, and attractive business proposition for some and a solution for others. In many affordable middle class or upper class families, even when there are relatives in good health who can donate, the general argument that is often presented is “why donate and take any risks when you can buy a kidney?” Organ trade in India like other problems such as child labor and prostitution has a societal issue to it. It relates to the exploitation of the poverty-stricken people by alluring them with financial gains that at times can be large and can meet their immediate short-term financial needs. Unlike other similar exploitative social situations, organ donation requires an invasive surgical procedure that has both physical and psychological implications.

The more recent live liver donation program has also been influenced by kidney donation and unrelated living donations have been reported in the media including two deaths.[6] Although kidney donation is a relatively safe surgery, the rising incidence of diabetes and hypertension in India makes the young donors potentially risk their health in the long-term. In some of the studies, it has been noted that when the motive of donation has been purely commercial, donors in the post-operative period have been more prone to ill-health. Whereas when the donation was purely altruistic, there was the feel-good factor and the psychological recovery was much better. In an interesting field study on Economic and Health Consequences of Selling a Kidney in India, it was found that 96% of participants (over 300) sold their kidneys to pay off debts. The average amount received was $1070. Most of the money received was spent on debts, food, and clothing. The average family income declined by one-third after removal of the kidney (p<.001) and the number of participants living below the poverty line increased. A total of three-fourths of the participants were still in debt at the time of the survey. About 86% of participants reported deterioration in their health status after nephrectomy. A total of 79% would not recommend that others sell a kidney. The article concludes that among the paid donors in India, selling a kidney does not lead to a long-term economic benefit and may be associated with a decline in health. Goyal, et al. conclude that: “In developing countries like India, potential donors need to be protected from being exploited. At a minimum, this might involve educating them about the likely outcomes of selling a kidney”.[7]

Lawrence Cohen, an anthropologist from Berkeley, interviewed patients in India and like Goyal had found that most of the donors were women who were deeply in debt and most of the money was squandered by their husbands in gambling and debts and the promise of a better future was never realized. In his research, Cohen found one-way trade in some of the “kidney belt region” of southern India where he investigated the trade route from organ sellers - usually poor rural women - to hospitals and recipients - often wealthy people from Sri Lanka and Bangladesh or from the Gulf States. Cohen found that poor people sold their kidneys to get out of debt or to support their families; yet most of these families were back in debt very shortly minus their kidneys. Some of the donors when asked if they would do it again said: ‘I'd do it again. I have a family to support. What choice did I have?’ Cohen states: “In some neighborhoods, the structure of debt appeared to rest on kidney selling since lenders would advance money knowing the organs were collateral. Moreover, there was no follow-up care after the operation nor were there efforts to prevent infection in the donor”.[8] When kidney donation is used as an option for quick financial gain, many donors do not realize that like any other major surgery it takes time to recoup health and has a certain amount of inherent risks. In their enthusiasm to get the money, they are somewhat blinded to all the explanations given about the surgery.

Giving in to market forces and making organs a commodity is fraught with dangers and erodes social, moral, and ethical values and is not an alternative that can be acceptable to overcome the problem of organ shortage in a civilized society. In her article on ‘The End of the Body: The Global Traffic in Organs for Transplant Surgery’, Nancy Scheper-Hughes, an anthropologist from Berkeley, states that by their very nature markets are indiscriminate, promiscuous, and inclined to reduce everything, including human beings, their labor and even their reproductive capacity to the status of commodities, to things that can be bought, sold, traded, and sometimes even stolen. Mr. Soros, the self made billionaire and a great believer of market forces, is deeply concerned with the erosion of social values and the dominance of anti-social market forces in the field of health sciences. He is of the opinion that a market economy is generally a good thing but opines that we cannot live by markets alone. Open and democratic societies require strong social institutions to serve such goals as social justice, political freedom, bodily integrity, and other human rights. The real dilemma, as Mr. Soros sees it, is one of uneven development. The evolution of the global market has outstripped the development of a mediating global society.[9]

The Bellagio Task Force from the Department of Anthropology, University of California, Berkeley with support from the Open Society Institute (from the Soros Foundation) conducted ethnographic research in sites in Brazil, India, and South Africa between 1997 and 1998. Their findings were as follows:

1. Strong and persistent race, class, and gender inequalities and injustices exist in the acquisition, harvesting, and distribution of organs
2. Violation of national laws that prohibit the sale of organs
3. The collapse of cultural and religious sanctions against body dismemberment and commercial use exist in the face of the enormous market pressures in the transplant industry
4. The appearance of new forms of traditional debt peonage in which the commodified kidney occupies a critical space
5. Persistent and flagrant human rights violations of cadavers in public morgues exist, with organs and tissues removed without any consent for international sale
6. The spread and persistence of narratives of terror concerning the theft and disappearance of bodies and body parts exists globally

Another report on The Global Traffic In Human Organs: a report presented to the House Subcommittee on International Operations and Human Rights, United States Congress on June 27, 2001 states that: “The growth of medical tourism for transplant surgery and other advanced procedures has exacerbated older divisions between the North and South and between the haves and have-nots. In general, the flow of organs, tissues, and body parts follows the modern routes of capital: from South to North, from third to first world, from poor to rich, from black and brown to white, and from female to male bodies. In the very worst instance, this market has resulted in theft and coercion, as in the case of China, to a self-serving belief in rights of the rich to the “spare parts” of the poor, as in the case of the many transplant junkets arranged to carry affluent patients from Saudi Arabia, Israel, and North America to Turkey, India, Romania, and the Philippines where kidney sellers are recruited from prisons, unemployment offices, and urban shantytowns.”

In an editorial in the New England Journal of Medicine, Francis Delmonico states that “The fundamental truths of our society, of life and liberty, are values that should not have a monetary price. These values are degraded when a poor person feels compelled to risk death for the sole purpose of obtaining monetary payment for a body part. Physicians, whose primary responsibility is to provide care, should not support this practice. Furthermore, our society places limits on individual autonomy when it comes to protection from harm. We do not endorse as public policy the sale of the human body through prostitution of any sort, despite the purported benefits of such a sale for both the buyer and the seller.”[10]

Cantarovitch suggests that organ transplantation depends on a social contract and social trust and it requires national and international law protecting the rights of both organ donors and organ recipients.[11] In the last few years, a group of physicians and policy makers in India have wanted to look at the possibility of making kidney sale a legal transaction by setting up some mechanism to protect them from middle men or brokers as it is being done in countries like Iran. These policy-makers should remember that the value of using short-term financial gains for donors to increase the supply of organs for transplantation is not a cure for poverty. As long there are people who can be exploited for money in society, certain evils are likely to perpetuate and legalizing the organ donation process will add another dimension to that evil and further weaken the social fabric.

### Organ shortage: A Global Issue

The high demand and poor supply of kidneys in the United States has widen over the years. This has resulted in many patients traveling abroad for transplant surgery. Some of the countries that have weak regulatory mechanisms have given in to the market forces and include India, Iran, China, Pakistan, Philippines, Brazil, Turkey, Moldova, Ukraine, Russia, Bulgaria, and Romania.

The World Health Organization (WHO) in its statement on the sale of organs clearly states that it violates the Universal Declaration of Human Rights as well as its own constitution: “The human body and its parts cannot be the subject of commercial transactions. Accordingly, giving or receiving payment… for organs should be prohibited.” The WHO advices physicians not to transplant organs “if they have reason to believe that the organs concerned have been the subject of commercial transactions.”[12]

More recently, the representatives of the world transplant community met in Istanbul to discuss the growing transplant donation commerce and transplant tourism. It defined ‘Transplant commercialism’ as a policy or practice in which an organ is treated as a commodity, including by being bought or sold or used for material gain.[13] In an editorial in Lancet on the subject, it says that “The success of transplantation as a life-saving treatment does not require—nor justify victimizing the world's poor people as the source of organs for the rich.”[14]

In India, where the deceased donation rate is abysmally small, there is a need to seriously explore this option and seems to be the way forward to our problem of organ shortage and to curb commerce in organs. Besides this swap or donor exchange in living transplants should be explored as a feasible alternative.

## SCOPE OF DECEASED DONATION PROGRAM

There are currently over 120 transplant centers in India performing approximately 3,500 to 4,000 kidney transplants annually. Out of these transplant centers, four centers undertake approximately 150 to 200 liver transplants annually while some of these centers also do an occasional heart transplant. Presently, approximately 50 liver transplants are done from deceased donors and the rest are from living donors. So far, 100 heart transplants have also been done.

In 1998, India had 1% of the world's road vehicles and 6% of the world's road accidents.[15] These accidents have increased to 10% in 2006. The total number of road accidents is approximately 90,000 per annum and in 2005 Tamil Nadu alone reported 13,000 fatal deaths due to road accidents.[16] In nearly 40–50% of all fatal road accidents in the world, the cause of death is head injury leaving potential organ donors in India from road traffic accidents alone. Other causes of brain death such as sub-arachnoids' hemorrhage and brain tumors would potentially add more numbers. Even if 5% to 10% of all these deceased patients became organ donors, it would mean that there would be no requirement for a living person to donate an organ. Promoting the deceased donation program would not only help kidney transplants but also liver, heart, pancreas, and lung transplants to thrive in the country.

There have been pockets of success with the deceased donation program and organ sharing among various hospitals. Five hospitals in Tamil Nadu and approximately eight hospitals in Hyderabad from the year 2000 to 2008 have successfully shared over 450 organs (170 organs in Hyderabad and 280 in Tamil Nadu) under the Organ Sharing Network that was initiated by a Non-Governmental Organization (NGO) called MOHAN Foundation.[17,18] In the past, Gujarat has had considerable success with the eye donation program due to the large population of the Jain community in the state. This community considers eye donation as a sublime form of charity and believes in a powerful link between ‘daan’ (charity) and ‘moksha’ (salvation). More recently, there has been a spurt of deceased solid organ donations in the state making deceased donation a possible alternative to the living transplant program. If properly organized, the deceased organ donation program has the potential to take care of the majority of the demands of kidney, liver, and heart transplants of that state.

Understanding the ethics of organ donation is important if we are to tackle the moral and ethical challenges that are emerging with cutting edge regenerative medicine such as stem cells transplants, cloning, and tissue re-engineering. The ethical principles of organ donation is an acid test that will help us in evolving and resolving many of the future moral issues that we are likely to encounter.

## CONCLUSION

The THO act despite having been passed 15 years ago has neither curbed commerce in organs nor helped promotion of the deceased donation program to take care of the organ shortage. The gap between the numbers of organs available and the number of patients joining the waiting list for a kidney transplant is widening globally. The high demand of organs has led to its commodification, more so in countries where there is a large proportion of the population below the poverty line with weak regulatory authorities. The resulting transplant tourism has caused an outcry from many international bodies. In India, the potential for deceased donation is huge due to the high number of fatal road traffic accidents and this pool is yet to be tapped. Few hospitals and committed NGOs in the country have shown that deceased donation as a feasible option. The ethics of kidney donation has important bearings on the society as this would form the basis to resolve many conflicts in emerging regenerative sciences.
